# New Aspects of the Antioxidant Activity of Glycyrrhizin Revealed by the CIDNP Technique

**DOI:** 10.3390/antiox11081591

**Published:** 2022-08-17

**Authors:** Aleksandra A. Ageeva, Alexander I. Kruppa, Ilya M. Magin, Simon V. Babenko, Tatyana V. Leshina, Nikolay E. Polyakov

**Affiliations:** 1 Voevodsky Institute of Chemical Kinetics and Combustion, 630090 Novosibirsk, Russia; 2 International Tomography Center, 630090 Novosibirsk, Russia

**Keywords:** glycyrrhizin, antioxidant activity, free radicals, solvated electron, photo-CIDNP, naproxen, 5-sulfosalicylic acid

## Abstract

Electron transfer plays a crucial role in ROS generation in living systems. Molecular oxygen acts as the terminal electron acceptor in the respiratory chains of aerobic organisms. Two main mechanisms of antioxidant defense by exogenous antioxidants are usually considered. The first is the inhibition of ROS generation, and the second is the trapping of free radicals. In the present study, we have elucidated both these mechanisms of antioxidant activity of glycyrrhizin (GL), the main active component of licorice root, using the chemically induced dynamic nuclear polarization (CIDNP) technique. First, it was shown that GL is capable of capturing a solvated electron, thereby preventing its capture by molecular oxygen. Second, we studied the effect of glycyrrhizin on the behavior of free radicals generated by UV irradiation of xenobiotic, NSAID—naproxen in solution. The structure of the glycyrrhizin paramagnetic intermediates formed after the capture of a solvated electron was established from a photo-CIDNP study of the model system—the dianion of 5-sulfosalicylic acid and DFT calculations.

## 1. Introduction

**Figure 1:**
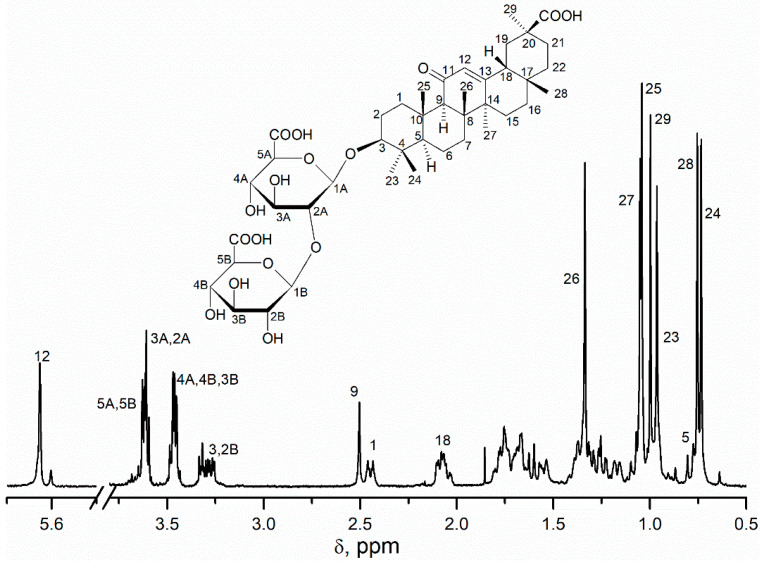
The structure and ^1^H NMR spectrum (500 MHz) of GL in D_2_O at pH = 7.2.

Glycyrrhizin (GL, Figure 1), the main active component of Licorice root (*Glycyrrhiza glabra* and *G. uralensis*), is one of the most frequently used drugs in traditional Persian, Chinese, and Japanese medicine. Since ancient times, licorice was employed to treat cough and asthma, lung, chest, liver, stomach, intestine diseases, indigestion, arterial diseases, urinary tract infections, kidney pain, the expulsion of kidney stones, ulcers, bladder diseases, fever, and eye diseases [1,2,3,4,5]. Recent studies also illustrated the significant effects of licorice root extract and glycyrrhizin on coronaviruses (including SARS-CoV-2) along with other viruses (herpes viruses, flaviviruses, hepatitis C virus, and influenza virus) [6,7,8,9,10,11,12,13,14,15,16,17,18].

However, increased interest in glycyrrhizin has recently been caused not only by its biological activity but also by its ability to enhance the effect of other medicinal compounds by forming supramolecular complexes with them [19]. During the last decades, novel unusual properties of glycyrrhizin have been discovered. It was demonstrated that due to its amphiphilic nature, glycyrrhizin is able to form self-associates in aqueous solutions, as well as water-soluble inclusion complexes with a variety of drugs [19,20,21,22,23,24,25]. Glycyrrhizin complexes significantly enhance drug solubility and bioavailability, so they are considered perspective drug delivery systems [19]. Furthermore, the membrane-modifying ability of glycyrrhizin has been demonstrated [26,27,28,29,30,31,32]. In particular, molecular dynamics simulation shows a significant decrease in the energy barrier of drug penetration through the lipid bilayer in the presence of glycyrrhizin in model lipid membranes [31,32]. This effect was explained by forming hydrogen bonds between drug and glycyrrhizin molecules in the middle of the bilayer.

This compound has excellent perspectives in combined therapy because of the biological activity of glycyrrhizin and its ability to work as a drug delivery system. As an example, the authors of a review [20] noted such a possibility in cancer therapy. They indicated that the combination of glycyrrhizin and first-line drugs had better therapeutic effects on cancers. Glycyrrhizin shows a series of antitumor-related activities, such as broad-spectrum anticancer ability, resistance to the tissue toxicity caused by chemotherapy and radiation, anti-multidrug resistance mechanisms [19,20], and drug absorption-enhancing effects as a carrier in drug delivery systems [31,32]. Furthermore, GL has a great capability to be conjugated with other anticancer molecules to synergistically enhance their combined cytotoxicity. On the other hand, some studies support the claim that glycyrrhizin has the potential to attenuate the side effects of anticancer drug overdose [33].

Another promising aspect of GL is antioxidant activity [34,35,36,37,38]. Considering the participation of antioxidants in various processes of living systems, antioxidant activity might be an essential feature of glycyrrhizin in the combined therapy of various diseases. It should be emphasized that, despite the abundance of examples of the antioxidant activity of glycyrrhizin in vivo and in vitro, there is still no consensus among scientists about the molecular mechanism of this activity of GL. Furthermore, discussions on this topic continue today [39,40,41,42,43,44,45,46,47,48,49]. Thus, some authors argue that glycyrrhizin does not scavenge hydroxyl radicals or superoxide anion radicals but scavenges 1,1-diphenyl-2-picrylhydrazyl (DPPH) radicals [41,42,43]. In contrast, other authors showed that glycyrrhizin is the scavenger of ROS radicals but does not scavenge DPPH radicals [44,45,46,47,48,49]. Thus, the authors of [46] measured the relative scavenging rate constants toward OOH radicals by glycyrrhizin and some carotenoid antioxidants using the EPR spin-trapping technique and showed that the antioxidant capacity of glycyrrhizin is even higher than that of beta-carotene and zeaxanthin. Pulse radiolysis study [47] indicated that glycyrrhizin offered radioprotection by scavenging free radicals and solvated electrons. The authors measured rate constants for the reaction of glycyrrhizin with OH radical and e_aq,_ which were 1.2 × 10^10^ M^−1^s^−1^ and 3.9 × 10^9^ M^−1^s^−1^, respectively. Radioprotection and photoprotection features of glycyrrhizin might be necessary for practical use in the defense of living tissues. The authors of [40] performed a computational investigation of O-H bond dissociation enthalpies and ionization potential of glycyrrhizin using the functional theory method and showed that its antioxidant character is correlated with the H-atom transfer mechanism.

In the present study, we have applied the chemically induced dynamic nuclear polarization (CIDNP) technique to shed light on the mechanism of the antioxidant activity of GL. CIDNP is one of the most informative indirect experimental methods for studying free radicals’ fate in complex chemical and biochemical processes [50,51,52]. In this work, we have traced the effect of glycyrrhizin on the behavior of paramagnetic particles generated by UV irradiation of xenobiotics, including drug molecules, in solutions. Furthermore, the radical intermediates of GL formed during its interaction with paramagnetic forms of xenobiotics have been elucidated using the analysis of the CIDNP effects and DFT calculation.

## 2. Materials and Methods

Naproxen (MP Biomedicals, 99.3%), 5-Sulfosalicilic acid hydrate (Aldrich Chem. Co., Milwaukee, WI, USA, 99%), glycyrrhizic acid (Shaanxi Pioneer Biotech Co., Tongchuan, China, 98%), and the deuterosolvents acetonitrile-d3 (99.5% D, Sigma, Burlington, MA, USA) and water-d2 (99.9% D, Cambridge Isotope Laboratories, Andover, MA, USA) were used as received. A Bruker Avance HD III NMR spectrometer (500 MHz ^1^H operating frequency, τ(π/2) = 10 μs) was used to record the ^1^H NMR spectra of the photoproducts. A Bruker DPX200 NMR spectrometer (200 MHz ^1^H operating frequency, τ(π/2) = 2.5 μs) was utilized to study the CIDNP effects. The Lambda Physik EMG 101 MSC excimer laser was used as a light source (308 nm, 100 mJ, pulse duration 15 ns) in these experiments. The samples were bubbled with argon for 15 min to remove dissolved oxygen and were irradiated directly in the probe of the DPX200 NMR spectrometer. Time-resolved (TR) CIDNP experiments were performed with the following pulse sequence: presaturation, laser pulse (−15 ns), variable time delay, and π/2 radio-frequency registration pulse.

Optimization of the structures of radical anion and neutral radical of glycyrrhizin and calculation of spin density distribution in these radicals were performed by density functional theory (DFT) calculations in vacuo, using PBE functional [53] and the quantum-chemical program PRIRODA [54,55] with L1 basis [56].

## 3. Results and Discussion

First, the effect of GL on radical particles formed during UV irradiation of the well-known anti-inflammatory drug naproxen (2-(6-methoxynaphthalen-2-yl) propanoic acid, NPX) in solutions was studied. The photolysis of NPX has been repeatedly investigated in connection with its known phototoxic side effects arising from both therapeutic uses and environmental degradation [57,58,59,60]. In general, phototoxic effects were associated with the appearance of radical species due to NPX photodegradation. Meanwhile, the structure and detailed mechanisms of the appearance of these particles have not been fully established. The introduction also mentioned that studies of the interaction of GL with ROS are not always detailed [41,42,43,44,45,46,47,48,49]. Therefore in this work, we used the CIDNP method, which allows us to trace the detailed mechanism of the appearance, structures, and interaction of radical species with each other. To the best of our knowledge, the time-resolved (TR) photo-CIDNP method was applied to study the effect of GL on the radical stages of NPX photodegradation for the first time.

The manifestation of CIDNP effects means the presence in the NMR spectra of products of radical reactions, carried out directly in the NMR spectrometer, signals with a non-Boltzmann population of nuclear spin projections: enhanced absorption or emission. The appearance of these signals in the NMR spectra results from the interaction of the spins of magnetic nuclei with the spins of electrons in a pair of radicals (RP)—precursors of the products. Several other parameters determine the phase and intensity of polarized signals, such as the multiplicity of RP (singlet or triplet), the signs of the HFI constant of RP partners and the difference in its g-factors, etc. The combination of these parameters determines the manifestation of emission or absorption on different groups of nuclei in the NMR spectra of the radical reaction products. An analysis of CIDNP spectra detected at a high magnetic field (as in our case) can be obtained by Kaptein’s well-known rules [61]. For the net effect, the CIDNP sign can be determined by the following parameters:

$$\Gamma_{i}=\left(\mu \right)\left(\varepsilon \right)\left(\Delta g\right)\left(a_{i}\right)$$

where *µ* denotes the initial spin multiplicity of radical pair (RP): “+” for triplet and F (RP from volume) and “−” for singlet RP, *ε* is the type of reaction leading to the observed products (“+” for cage product, “−” for escaped products), Δ*g* = (*g*_1_ − *g*_2_) is the sign of the difference in g-factors of the two radicals (*g*_1_ is the g-factor of the radical with nucleus under observation), *a*_i_ is the sign of the HFI constant of this radical. Thus, the final sign of integral polarization of nuclei “*i*” with these parameters in the product determines Γ*_i_*, indicating absorption (A) or emission (E). Magnetic resonance parameters of radicals are listed in Supplementary Materials (Table S2).

Analysis of polarized signals according to the above rules allows one to establish the structure of the paramagnetic precursors of the NPX products formed under UV irradiation in acetonitrile:water (1:3) solution in the presence and absence of GL. The assignment of the main products of NPX photolysis (2-(1-hydroxyethyl)-6-methoxynaphthalene and 2-ethyl-6-methoxynaphthalene) was carried out based on the reference data [57,58,59,60] (Table S1, Figures S1 and S2). It is worth noting that apart from the abovementioned photoproducts, several dimer structures were found.

**Figure 2:**
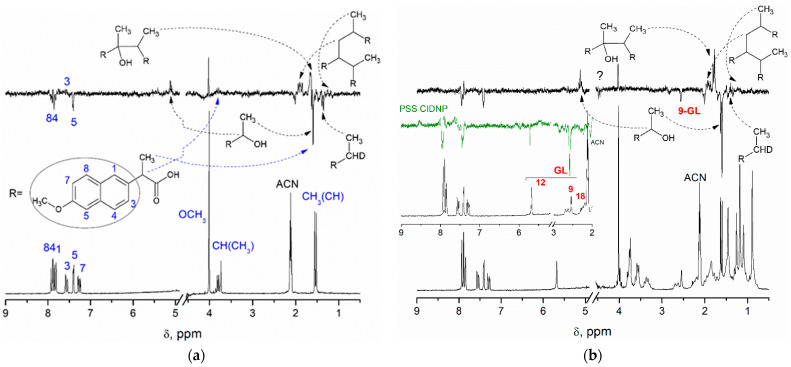
(**a**) ^1^H NMR and TR-CIDNP spectra of 4.3 mM NPX; (**b**) an equimolar mixture of NPX and GL (4.3 mM) in acetonitrile:water (1:2) solution; in insert: pseudo-steady-state (PSS) CIDNP spectra of NPX with GL under the same conditions (green). The delay time between the laser pulse and the NMR detection pulse is 0. Numbers designate the aromatic protons of NPX (blue) and GL (red).

The first example of NMR and CIDNP spectra detected upon UV irradiation of NPX in the absence and presence of GL is shown in Figure 2.

The analysis carried out according to Kaptein’s rules is presented in Table 1.

**Scheme 1:**
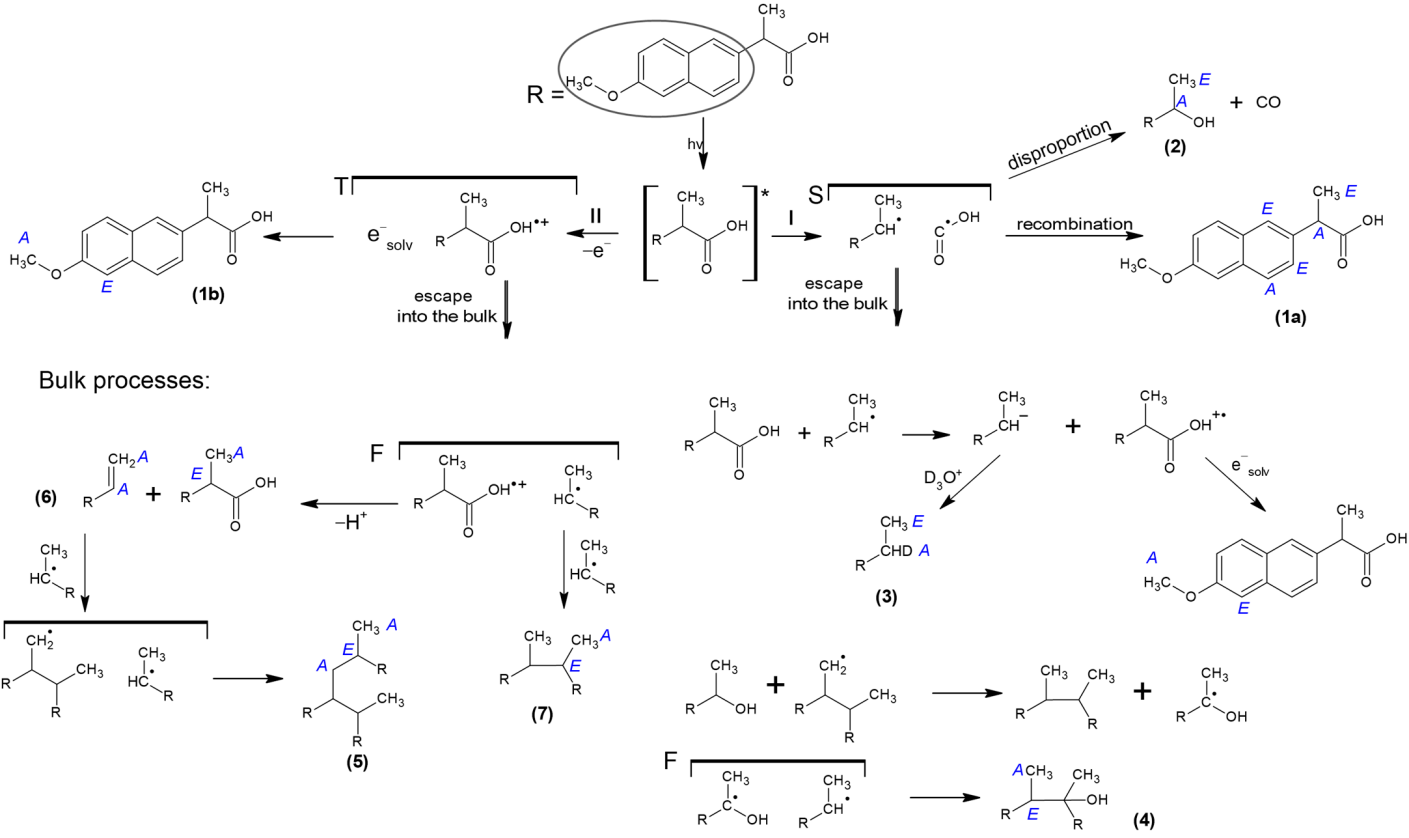
Photoinduced processes of NPX in the excited singlet state. Horizontal brackets represent radical pairs (RP)—precursors of products: S is primary singlet RP (geminal), T is RP formed from triplet excited state, F is RP formed when radicals are encountered in bulk. An asterisk denotes an excited state.

The above analysis results of the photo-CIDNP signs from Figure 2a, detected using various CIDNP measurements (TR and PSS), point to the following scheme of radical stages of the NPX photodegradation (Scheme 1). In this case, the reconstruction of the processes’ scheme is based on establishing the structures of radical precursors of polarized products. Scheme 1 summarizes two pathways (I and II) of product formations through the radical stages.

This scheme of NPX photolysis is also confirmed by the reference data, according to which NPX undergoes photoionization (path II in Scheme 1) [57]. CIDNP sign analysis shows that in the case of the second path, polarization in RP: NPX radical-cation and solvated electron is formed in the pair of radicals in the collective triplet spin state. Since CIDNP analysis of signs reveals only the prevailing path, it is impossible to trace the polarization of NPX from the excited triplet state. Nevertheless, this was possible in the presence of GL, the polarization of which is formed in only one way. In addition, it should be noted that the reaction of singlet oxygen with the triplet state of NPX is characteristic [57,60].

**Figure 3:**
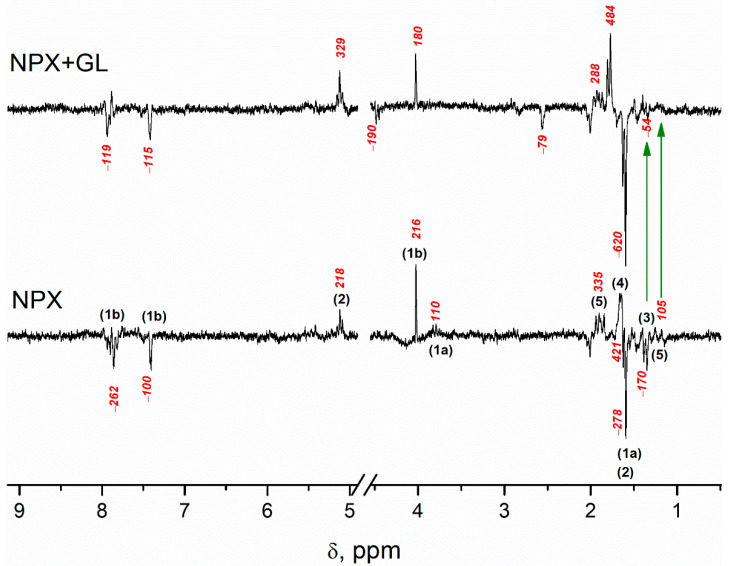
^1^H TR-CIDNP spectra of 4.3 mM NPX (bottom) and an equimolar mixture of NPX and GL (top) in acetonitrile: water (1:2) solution with integrals of signals (red). Assignment of the signals (black) corresponds to the structures of product numbering in Scheme 1. The delay time between the laser pulse and NMR detection pulse is 0 for both cases. Green arrows show the most pronounced effect of GL on the bulk products.

The influence of GL (the structural formula and the NMR spectrum are shown in Figure 1) on the CIDNP effects of NPX photolysis is shown in Figure 2b. In this case, we assume that at the concentrations used, GL should not form stable complexes with NPX. The comparison of the CIDNP spectra shown in Figure 2a,b compels the authors to believe that GL in this system might be an antioxidant, capturing a solvated electron to form a GL radical-anion and possibly acting as a trap for short-lived radicals presented in Scheme 1. Indeed, for the GL itself, we observe polarized 9-CH, 12-CH, and 18-CH protons (Figure 2b). The influence of GL as a trap can be seen by comparing the polarized lines’ intensity presented in Figure 2a,b, which is also quantitatively illustrated in Figure 3.

The effect of GL is revealed as a two-fold increase in CIDNP intensity of NPX methyl protons (1a), while according to the evaluation of the integrals, the bulk products (3) in the presence of GL are decreased by 20–30%. It results from the decrease in the yield of the radicals escaped into the bulk. It is worth emphasizing that the effect of GL on CIDNP of products formed in bulk, for example, in the case of (5), is comparable to that for initial NPX (green arrows in Figure 3 show the most pronounced effect of GL). The participation of GL in these processes is also confirmed by its polarization (see Figure 2b). The short-lived radicals shown in Scheme 1 are likely to extract terminal protons from GL or attach to the double bond of the acid.

**Figure 4:**
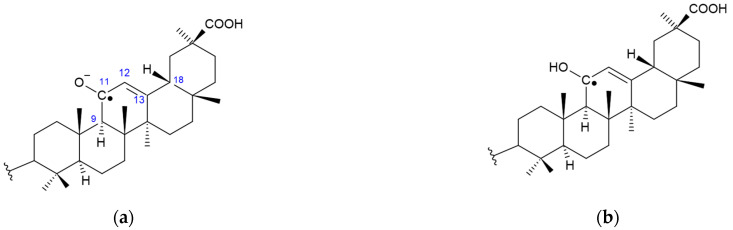
Two possible paramagnetic forms of GL. The glucuronide fragment of GL is omitted in the figure. (**a**) The radical anion of GL (R1), (**b**) ketyl-type radical of GL (R2).

It can be seen from Figure 2b that the strongest polarization on GL is observed on the 9-CH proton, which can only occur when the spin density is localized in the intermediate GL radical on the unsaturated C11-C12-C13 chain. In this case, two types of radicals can be represented: the GL radical-anion, formed upon the capture of a solvated electron by the GL molecule; and the ketyl-type radical. The latter can be obtained by protonating the primary radical anion (Figure 4).

Table 2 presents the calculated (DFT) values of the HFI constants in these radicals.

As can be seen from Table 2, 9-CH protons of both GL radicals have a prevailing HFI constant. It was previously determined that GL can capture a solvated electron with a rate constant of 3.9 × 10^9^ M^−1^s^−1^ [47], close to the diffusion limit in water, and suggests the formation of R1. Moreover, as described above, the NPX photolysis pathway includes the possibility of photoionization. As shown in Figure 2b, the polarization of one sign for all three polarized protons of the GL is observed. This indicates that the CIDNP is formed in the RP with the participation of R2 (Table 3).

**Scheme 2:**
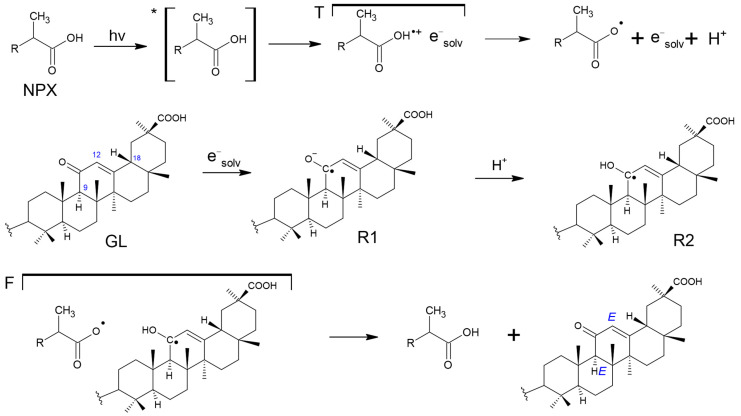
The formation of GL radical anion (R1) and its further transformation into R2 during NPX photolysis in the presence of GL. An asterisk denotes an excited state; T is RP formed from the triplet excited state.

In the case of NPX photolysis in the presence of GL, a possible way for R1 to transform into R2 is shown in Scheme 2.

Since NPX photolysis is a very complex process, we used a model reaction of two-quantum ionization of dianion of 5-sulfosalicylic acid to confirm the capture of a solvated electron by GL in an aqueous solution at pH = 10. In this case, GL does not form associates that can affect its interactions with a solvated electron.

**Figure 5:**
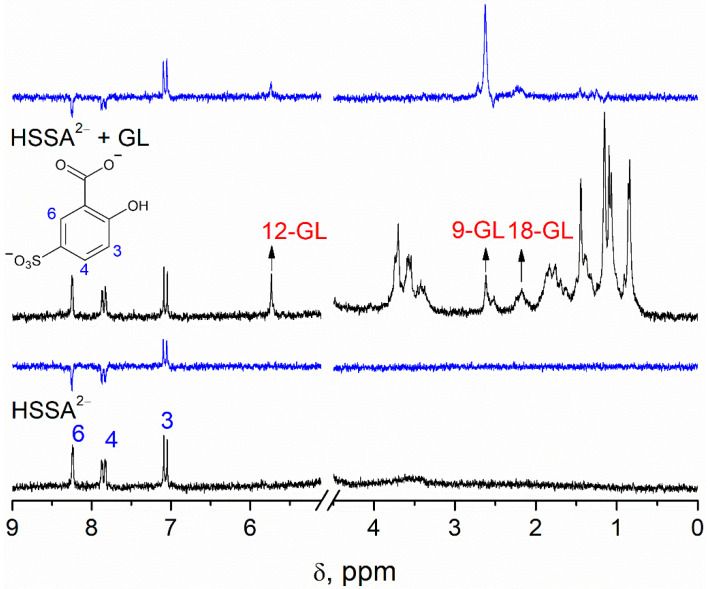
^1^H NMR and TR-CIDNP (blue) spectra of 1 mM HSSA^2−^ in water (pH = 10) and 1 mM HSSA^2−^ and 1.5 mM GL in water (pH = 10). The delay time between the laser pulse and NMR detection pulse is 50 μs for both cases.

**Scheme 3:**
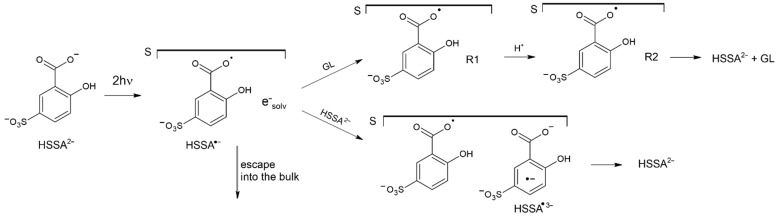
Possible photoinduced processes occurring during HSSA^2−^ photolysis in the presence of GL.

The photolysis of the dianion of 5-sulfosalicylic acid (HSSA^2−^) was previously investigated by TR-CIDNP and various optical methods with time resolution [63]. CIDNP was shown to be formed in the pair of ^1^[HSSA^•−^ e_aq_^−^] in two-quantum photoionization of HSSA^2−^. Figure 5 demonstrates the NMR spectra of HSSA^2−^ (1 mM) in the absence and presence of GL (1.5 mM) in aqueous solutions (pH = 10), as well as TR-CIDNP spectra observed upon photoexcitation of these solutions. Figure 5 clearly shows that the CIDNP pattern of HSSA^2−^ fully corresponds to the results of the previous investigation [63,64]. In this case, the presence of GL in the solution does not change the nature of the polarization of HSSA^2−^. However, GL demonstrates the polarization of 9-CH, 12-CH, and 18-CH protons with the same signs. HFC signs of 9-CH, 12-CH, and 18-CH in the R2 radical are positive; the g-factor of the ketyl radical (R2) is in the range of 2.0030–2.0032, and the g-factor of HSSA^•−^ = 2.00476 [63]. The CIDNP sign of GL protons (Γ > 0, A) corresponds to the cage recombination product of singlet RP (*ε* > 0, *μ* < 0, Δ*g* < 0). These findings suggest that polarization is formed in RP with R2 upon protonation of R1 (see Scheme 3).

The most probable transformations of the primary active intermediate particles in the system under study, that is, the recombination of the primary radical ion pair (RIP) with the formation of the initial HSSA^2−^ and the capture of the solvated electron by GL and HSSA^2−^ (with rate constants of 3.9 × 10^9^ M^−1^s^−1^ [47] and 2.7 × 10^9^ M^−1^s^−1^ [63]), are shown in Scheme 3.

This scheme corresponds to all the observed signs of CIDNP (Figure 5) in the model process of (HSSA^2−^) photoionization. It also shows the possibility of electron capture by GL with the formation of the radical anion R1 and its further transformation into the ketyl radical R2. The latter is especially important in reactions where there is no ionization of GL itself, but there are parallel pathways involving neutral radicals, the reactions of which can be affected by the presence of R2, as, for example, in the photolysis of NPX described above.

## 4. Conclusions

Thus, this work presents a systematic study of the interaction of GL with short-lived paramagnetic particles, including a solvated electron produced by UV irradiation of widely used NSAID—NPX and the model system—dianion of 5-sulfosalicylic acid (HSSA^2−^). An analysis of the CIDNP data has demonstrated a noticeable effect of GL on the concentration of radical particles formed during the photolysis of NPX. In particular, this effect manifests itself as an increase in the reversibility of photodecomposition (an increase in the CIDNP of the initial NPX) and a decrease in the CIDNP of radicals involved in bulk processes. In connection with the known toxicity of short-lived radicals, it can be expected that the inclusion of GL, for example, in the composition of ointments, can lead to diminishing drug toxicity. In addition, it was reliably established that the GL molecule captures a solvated electron generated under UV irradiation of both the NPX and the model system (HSSA^2−^). Finally, the paramagnetic forms of GL have been characterized by CIDNP data and DFT calculation.

## Figures and Tables

**Table 1:** Analysis of CIDNP signs of NPX products.

N	Compound	Group of Protons	*μ*	*ε*	Δ*g*	*a_i_*	Γ*_i_*
1a	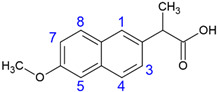	CH_3_	−	+	+	+	E
		CH	−	+	+	−	A
		1,3-CH	−	+	+	−	A
		4-CH	−	+	+	+	E
2	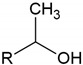	CH_3_	−	+	+	+	E
		CH	−	+	+	−	A
3	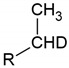	CH_3_	+	−	+	+	E
		CH	+	−	+	−	A
4	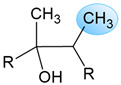	CH_3_	+	+	+	+	A
		CH	+	+	+	−	E
5	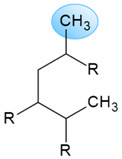	CH_3_	+	+	+	+	A *
		CH_2_	+	+	−	−	A *
6	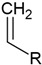	CH_2_	−	−	+	+	A **
		CH	−	−	+	+	A **
7	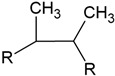	CH_3_	−	−	+	+	A **
		CH	−	−	+	−	E **

(*) the observed polarization is a superposition of the effects formed in S and F RPs from Scheme 1. (**) sign of nuclei polarization corresponds to the escape product from the primary (geminal) singlet RP.

**Table 2:** DFT calculation of HFI constants for two paramagnetic forms of GL.

Position	HFI Constants in R1, mT	HFI Constants in R2, mT
9-CH	+0.266	+0.676
12-CH	−0.069	+0.072
18-CH	+0.147	+0.206

**Table 3:** Analysis of CIDNP signs of NPX in the presence of GL.

Compound	Group of Protons	*μ*	*ε*	Δ*g*	*a_i_*	*γ* *	Γ*_i_*
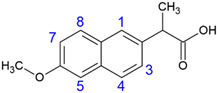	5-CH	+	+	+	−	+	E
	OCH_3_	+	+	+	+	+	A
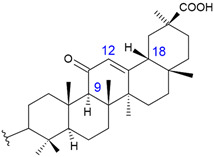	9-CH	+	+	−	+	+	E
	12-CH	+	+	−	+	+	E

(*) γ refers to the multiplicity of the radical ion pair (RIP) that recombines (+ for the recombination of RIP in singlet spin state, − for triplet), taking into account the possibility of RIP recombination from both spin states [62].

## Data Availability

Data is contained within the article.

## Supplementary Materials

The following supporting information can be downloaded at: https://www.mdpi.com/article/10.3390/antiox11081591/s1, Table S1: Products of NPX photodegradation from literature data in aqueous solutions under aerobic and anaerobic conditions Figure S1: ^1^H NMR spectra of NPX and its photodegradation products after photolysis in acetonitrile:water (1:2); Figure S2: ^1^H NMR spectra of NPX and its photodegradation products after photolysis in the absence and presence of GL in acetonitrile:water (1:2) solution. Refs. [65,66,67,68] is cited in Supplementary Materials.
