# Supplementary material for: New Aspects of the Antioxidant Activity of Glycyrrhizin Revealed by the CIDNP Technique
Source: Antioxidants (Basel). 2022 Aug 17;11(8):1591. doi: 10.3390/antiox11081591 (PMC9405345; doi:10.3390/antiox11081591)
Supplement: Supplementary file 1 [file antioxidants-11-01591-s001.zip › antioxidants-1840685-supplementary.pdf]

Supplementary Materials to

New aspects of the antioxidant activity of glycyrrhizin revealed by the CIDNP technique

Aleksandra A. Ageeva[1], Alexander I. Kruppa[1], Ilya M. Magin[1], Simon V. Babenko[1,2], Tatyana V. Leshina[1], Nikolay E. Polyakov[1]*

[1] Voevodsky Institute of Chemical Kinetics and Combustion, 630090 Novosibirsk, Russia;

[2] International Tomography Center, 630090 Novosibirsk, Russia

Corresponding author: polyakov@kinetics.nsc.ru

Table S1. Products of NPX photodegradation from literature data in aqueous solutions under aerobic and anaerobic conditions [1-2].

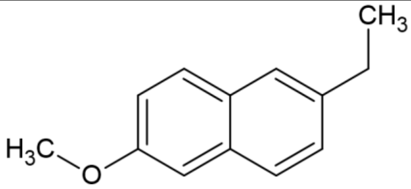	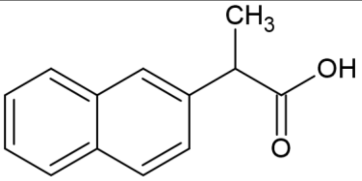
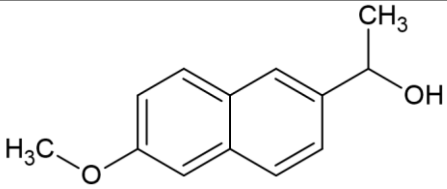	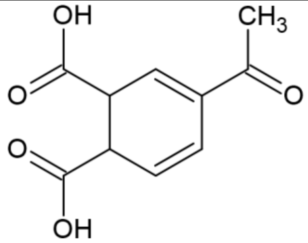
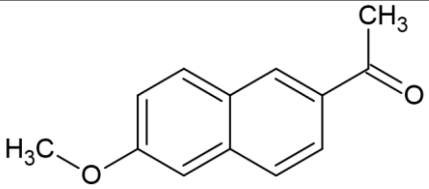	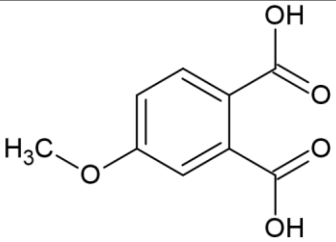
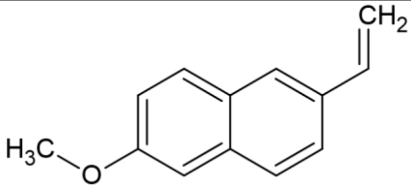	

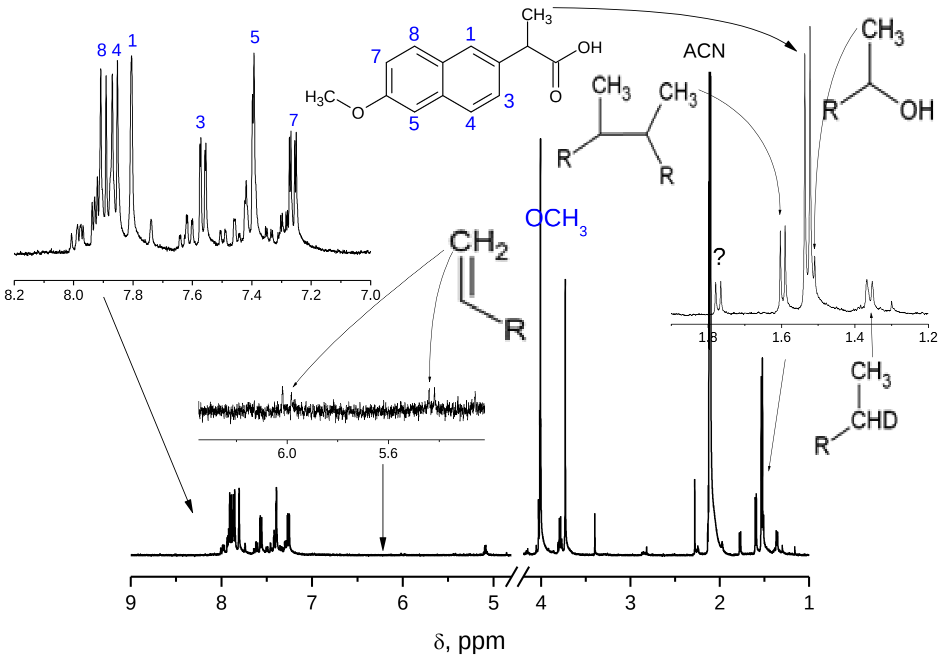

Figure S1. ^{1}H NMR spectra of NPX and its photodegradation products after photolysis in acetonitrile:water (1:2).

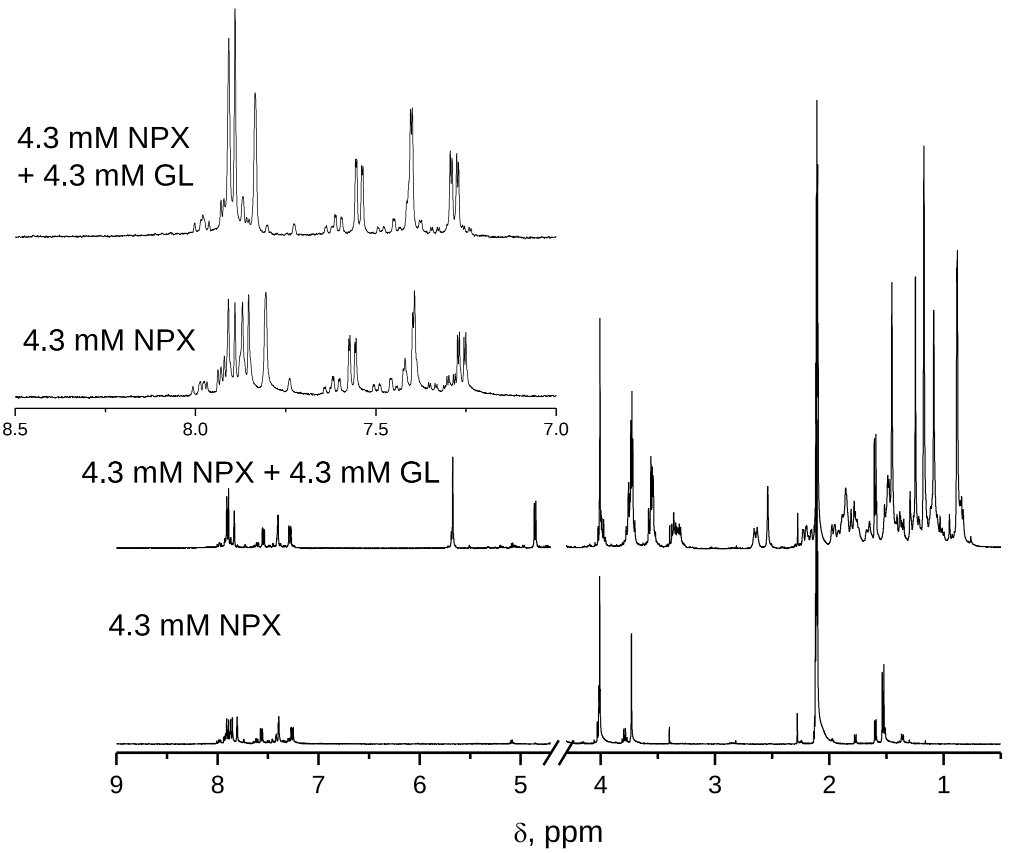

Figure S2. ^{1}H NMR spectra of NPX and its photodegradation products after photolysis in the absence and presence of GL in acetonitrile:water (1:2) solution.

Table S2. Magnetic resonance parameters of radicals.

Structure	g-factor/ a-value (mT)
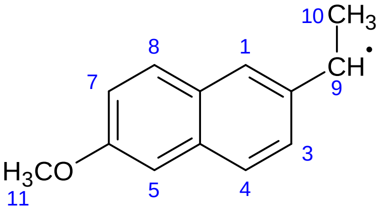	2.0027 [3]<br><br>3H (10): +1.770<br>H (9): -1.628<br>H (1,3): -0.512<br>H (4): +0.167
•COOH	2.0002 [4]
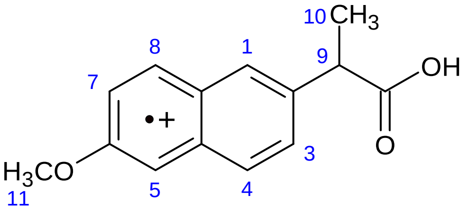	2.0032 [5]<br><br>3H (11): + 0.15<br>H (1,5): -0.20
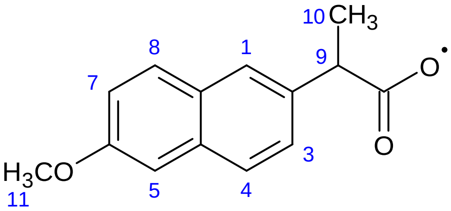	2.0058 [6]

References

1. Boscá, F.; Miranda, M. A.; Vañó, L.; Vargas, F. New Photodegradation Pathways for Naproxen, a Phototoxic Non-Steroidal Anti-Inflammatory Drug. *Journal of Photochemistry and Photobiology A: Chemistry* **1990**, *54* (1), 131–134. https://doi.org/https://doi.org/10.1016/1010-6030(90)87018-7
2. Cazzaniga, N.; Varga, Z.; Nicol, E.; Bouchonnet, S. UV-Visible Photodegradation of Naproxen in Water – Structural Elucidation of Photoproducts and Potential Toxicity. *European Journal of Mass Spectrometry* **2020**, *26* (6), 400–408. https://doi.org/10.1177/1469066720973412.
3. Berndt, A. 4.4.6.2 Benzyl Radicals with One Substituent at C(7): Datasheet from Landolt-Börnstein - Group II Molecules and Radicals · Volume 17C: ``Conjugated Carbon-Centered and Nitrogen Radicals" in SpringerMaterials (Https://Doi.Org/10.1007/10348248_49). Springer-Verlag Berlin Heidelberg. https://doi.org/10.1007/10348248_49.
4. Norman, R. O. C.; West, P. R. Electron Spin Resonance Studies. Part XIX. Oxidation of Organic Radicals, and the Occurrence of Chain Processes, during the Reactions of Some Organic Compounds with the Hydroxyl Radical Derived from Hydrogen Peroxide and Metal Ions. *Journal of the Chemical Society B: Physical Organic* **1969**, No. 0, 389–399. https://doi.org/10.1039/J29690000389.
5. Schaffner, E.; Kweton, M.; Vesel, P.; Fischer, H. Photoinduced Electron Transfer from Naphthalenes to Dicyanoethenes and Subsequent Radical Ion Processes Studied by Time Resolved CIDNP. *Applied Magnetic Resonance* **1993**, *5* (2), 127–150. https://doi.org/10.1007/BF03162517.
6. Kaptein, R.; Brokken-Zijp, J.; de Kanter, F. J. J. Chemically Induced Dynamic Nuclear Polarization. XI. Thermal Decomposition of Acetyl Peroxide. *J Am Chem Soc* **1972**, *94* (18), 6280–6287. https://doi.org/10.1021/ja00773a004.
